# Changes of urine isolates of *Escherichia coli* and *Klebsiella pneumoniae* biofilm affect monocytes’ response

**DOI:** 10.1007/s11274-021-03150-y

**Published:** 2021-09-28

**Authors:** Agnieszka Daca, Justyna Gołębiewska, Marek Bronk, Tomasz Jarzembowski

**Affiliations:** 1 Department of Pathology and Experimental Rheumatology, Medical University of Gdansk, Gdansk, Poland; 2 Department of Nephrology, Transplantology and Internal Diseases, Medical University of Gdansk, Gdansk, Poland; 3 Laboratory of Clinical Microbiology, University Clinical Centre, Gdansk, Poland; 4 Department of Microbiology, Medical University of Gdansk, Gdansk, Poland

**Keywords:** Monocytes, Biofilm, Adhesion, Immunosuppression, Kidney disease

## Abstract

The Gram negative rods as *Escherichia coli* and *Klebsiella pneumoniae* belong to the most common etiology agents of urinary tract infections. The aim of our study was to assess the diversity of biofilm formed in different urinary tract diseases and their impact on monocytes’ adherence and activation. The bacteria were obtained from patients with different kidney problems. Some of the patients were after renal transplantation, some of them were not. Changes in the size and granularity of monocytes, as well as their adherence to biofilm, were assessed using FACSVerse flow cytometer after 1 h co-incubation of monocytes and bacterial biofilm in 37 °C. The obtained results were validated against monocytes incubated without bacteria. The isolates from patients with chronic kidney disease formed the most adherent biofilm regardless the presence or absence of inflammatory reaction. Adherence of monocytes also increased during therapy with immunosuppressive agents, but monocytes’ response was different when cyclosporine or tacrolimus were used. Additionally the presence of inflammatory reaction in patients with kidney disease modified the monocytes response when the immunosuppressive drugs were used. Considering the obtained results, we conclude that the changes of monocytes’ morphology in response to biofilm formed by Gram negative rods could become a tool to detect urinary tract infection, especially in those groups of patients, where the knowledge of ongoing inflammation is important and the standard tools fail to detect it.

## Introduction

The dispute on differences between commensals found accidentally in urine and pathogens of urinary tract seems to be far from complete. Despite many efforts undertaken by researchers, the question how to estimate the risk of infection’s development remains unanswered. Urinary tract infection (UTI) is a serious health problem for several reasons, e.g. increasing problem with antibiotic resistance, possible renal scarring and sepsis development as a complication (Khan et al. 2019; Sabih and Leslie 2020). Additionally the recurrence rate is high and often the infections tend to become chronic with many episodes. From the other hand, asymptomatic bacteriuria occurs in up to 20% of healthy individuals (Hancock et al. 2007; Ejrnæs et al. 2011).

The most common etiology agents of UTIs are Gram negative rods of Enterobacteriaceae family, including *Escherichia coli* and *Klebsiella pneumoniae*. Because of the common antibiotic resistance of *K. pneumoniae*, infections caused by this pathogen are raising concern (Gołębiewska et al. 2019). *K. pneumoniae* may cause both, community-acquired and hospital-acquired infections. Isolates from hospital-acquired infections usually lack virulence genes but are resistant to many antibiotics. *K. pneumoniae* has been also shown to be the most common pathogen in recurrent UTIs during the first year after renal transplantation (Gołębiewska et al. 2019)**.**

*Escherichia coli*, the same as *K. pneumoniae,* can cause both nosocomial and community-acquired infections. Many researches so far proven extreme fitness of those bacteria, which of course relate to the commonness of this pathogen as a virulence factor of UTI. Among many properties, the ability to survive in limited iron availability, or the ability to speed up metabolism in relatively scanty in nutrients environment increase the fitness of this bacteria (Subashchandrabose and Mobley 2016). It was also discovered that *E. coli* can invade and replicate within bladder, forming biofilm-like intracellular bacterial communities (IBCs) and establish quiescent intracellular reservoirs that may represent stable environment for RUTI (recurrent UTI) (Ejrnæs et al. 2011). That fact in itself can explain, why about 77% of RUTIs are caused by the same strain (Ejrnæs et al. 2011).

It becomes also more and more clear that the properties of biofilm formed by bacteria rather than the properties of planktonic cells are the key to proper diagnosis (Ejrnæs et al. 2011; Jarzembowski et al. 2018). The problems with recurrence of UTI and resistance to treatment are directly keyed to the resistance of bacterial biofilm to standard forms of eradication.

Monocytes are, directly after neutrophils, the first appearing at the site of infection. Their number, but also the biochemical activity will be important when it comes to the effective elimination of the bacteria. The main mechanism utilized by those cells to fight off an offending agent is of course phagocytosis and subsequent oxidative burst. Both of them depend on basic cellular processes. Phagocytosis requires specific cytoskeleton rearrange (Mancilla-Herrera et al. 2015), oxidative burst is based on the production of many enzymes, such as myeloperoxidase, hydrolases and elastases, lysozyme and proteases (Koenig et al. 2017). Both parameters can be assessed using the flow cytometer. The changes in cytoskeleton will be reflected by the changes in the size of the cell and flow cytometric FSC (forward scatter) parameter. The increased protein—enzymes—production will be reflected in the cell’s changes in granularity and SSC (side scatter) parameter. The exposure of monocytes to biofilm formed by bacteria isolated from urine of the patients with kidneys diseases will also cause abovementioned changes but they can be different depending not only on the features of bacteria but also on the condition of immune system cells.

To simplify the observed interactions, here we exposed human cell line monocytes (THP-1) to biofilm and assessed the differences in their response in the presence or absence of specific agents, such as e.g. the presence/absence of inflammatory reaction or the presence/absence of specific immunosuppressive drugs in treatment protocol. The fact that specific comorbidities seem to additionally affect the selection of strains causing RUTI is the reason, why we aimed to evaluate the influence of other clinical parameters of the host on the biofilm properties of urine isolates of *E. coli* and *K. pneumoniae.* To analyze biofilm properties, we exposed monocytes on biofilm cells. Their response seems to visibly differ, depending the bacteria features in the presence of different comorbid diseases.

## Materials and methods

### Patients

Group of patients include 68 renal transplant recipients (RTx) and 76 patents without kidney transplantation from Nephrology, Transplantology and Internal Diseases Department of University Clinical Centre, Gdansk. 98 urinary cultures of *E. coli* and 46 urinary cultures of *K. pneumoniae* were collected from them. The full characteristic of patients is presented in Table 1.

**Table 1:** Characteristic of patients

	No [W/M]*	Age [mean ± SD]	Immunosuppressive therapy	Underlying disease: no [W/M]	eGFR [mean ± SD]	Creatinine concentration	CRP	UTI presence [Y/N]***
All	144 [97 / 47]	50.72 ± 24.45 W 50.65 ± 25.51 M 50.87 ± 22.97	Tacrolimus: 59 Cyclosporine: 4 Other: 15 None: 66	ADPKD: 10 [8/2]	36.90 ± 12.29	1.93 ± 0.94	87.32 ± 94.45	9/1
				Glomerulonephritis: 9 [3/6]	46.00 ± 32.06	1.62 ± 0.79	34.60 ± 48.28	8/1
				Nephropathy**: 10 [3/7]	18.80 ± 6.88	2.89 ± 0.75	34.60 ± 48.28	7/3
				Recurrent UTI: 8 [5/3]	49.125 ± 30.27	1.67 ± 0.48	108.12 ± 150.22	5/3
				SLE: 7 [7/0]	38.28 ± 27.38	1.94 ± 0.70	11.07 ± 7.58	5/2
				Nephrotic syndrome: 5 [3/2]	25.66 ± 37.20	5.50 ± 0.00	26.58 ± 41.85	0/5
				Chronic kidney disease^#^: 20 [18/2]	34.88 ± 18.70	3.86 ± 7.51	41.15 ± 56.65	15/5
				None: 11 [8/3]	61.00 ± 36.32	1.34 ± 0.78	93.27 ± 85.78	9/2
				Others: 64 [38/26]	51.89 ± 34.62	2.31 ± 1.66	70.22 ± 98.32	42/22
RTx patients	68 [44 / 24]	51.09 ± 18.16 W 48.43 ± 20.20 M 55.96 ± 12.20	Tacrolimus: 58 Cyclosporine: 4 Other: 6 None: 0	ADPKD: 10 [8/2]	36.90 ± 12.29	1.93 ± 0.94	87.32 ± 94.45	9/1
				Glomerulonephritis: 9 [3/6]	46.00 ± 32.06	1.62 ± 0.79	34.60 ± 48.28	8/1
				Nephropathy**: 0 [0/0]	–	–	–	–
				Recurrent UTI: 4 [2/2]	33.00 ± 0.00	2.00 ± 0.00	2.30 ± 0.00	4/0
				SLE: 5 [5/0]	26.80 ± 12.35	2.21 ± 0.61	15.40 ± 5.40	3/2
				Nephrotic syndrome: 0 [0/0]	–	–	–	–
				Chronic kidney disease^#^: 15 [13/2]	28.86 ± 8.25	4.27 ± 7.95	36.37 ± 57.90	12/3
				None: 0 [0/0]	–	–	–	–
				Others: 25 [13/12]	41.62 ± 29.39	2.19 ± 1.46	72.85 ± 88.22	14/11
Non-RTx patients	76 [49 / 27]	50.39 ± 28.95 W 52.44 ± 28.21 M 45.37 ± 30.11	Tacrolimus: 1 Cyclosporine: 0 Other: 9 None: 66	ADPKD: 0 [0/0]	–	–	–	–
				Glomerulonephritis: 0 [0/0]	–	–	–	–
				Nephropathy**: 10 [3/7]	18.80 ± 6.88	2.89 ± 0.75	34.60 ± 48.28	7/3
				Recurrent UTI: 4 [3/1]	65.25 ± 36.23	1.005 ± 0.145	161.04 ± 59.53	1/3
				SLE: 2 [2/0]	67.00 ± 33.00	1.26 ± 0.36	2.40 ± 1.24	2/0
				Nephrotic syndrome: 5 [3/2]	25.66 ± 37.20	5.50 ± 0.00	26.58 ± 41.85	0/5
				Chronic kidney disease^#^: 5 [5/0]	63.00 ± 26.55	0.99 ± 0.31	72.20 ± 34.05	3/2
				None: 11 [8/3]	61.00 ± 36.32	1.34 ± 0.78	93.27 ± 85.78	9/2
				Others: 39 [25/14]	57.95 ± 36.02	2.41 ± 1.80	68.41 ± 104.67	28/11

*W—women, M—men

**Diabetic nephropathy and hypertensive nephropathy

***Y—yes, N—no, asymptomatic bacteriuria was not considered as UTI

#chronic kidney disease with unknown aetiology

The RTx patients initially underwent induction with monoclonal (basiliximab) or polyclonal antibodies (ATG) and were prescribed subsequently TAC (tacrolimus) + MMF (mycophenolatemofetil)/MPS (mycophenolate sodium) + glucocorticosteroids or CsA (cyclosporine) + MMF/MPS + glucocorticosteroids or CsA + everolimus + glucocorticosteroids.

The work described here has been carried out in accordance with Declaration of Helsinki. The Local Independent Committee for Ethics in Scientific Research at Medical University of Gdansk reviewed and approved the experiment protocol and outline (NKBBN/504-71/2018). All of patients, from whom the material was obtained, gave their written informed consent.

### Bacterial culture

The isolates were identified to species level by strep ID test (BioMerieux, France) and classified as different strains of *E. coli* and *K. pneumoniae* by biochemical and resistance profiles. All bacterial strains were stored at (-70 °C) in brain heart infusion (BHI) broth with 25% (vol/vol) glycerol. Biofilms of these strains were obtained as described earlier (Jarzembowski et al. 2018), briefly by culturing at 37 °C on flat-bottom 6-wells plates (TRP, Switzerland) for 72 h in BHI medium. After another 28 h, medium was replaced with fresh 2 ml of BHI.

### Monocytes activation assay

The procedure evaluated during our previous study was applied (Jarzembowski et al. 2018). As a reference, monocytes THP-1 cell line (TIB-202, ATCC, USA) was used. The cells were cultured in RPMI-1640 medium supplemented with 2 mM L-glutamine, 100U/ml penicillin, 100 µg/ml streptomycin and 10% (vol/vol) heat-inactivated fetal bovine serum (FBS) (all from Sigma-Aldrich, Denmark).

Wells with biofilm were washed with 0,9% NaCl; suspension of monocytes (2,5*10^5^ of cells per well) was then added and incubated at 37 °C for 60 min on orbital shaker. Sterile wells were used as reference wells for adhesion and activation of monocytes.

For evaluation of monocytes’ activation, the number of monocytes and their morphology (described by FSC—forward scatter parameter featuring the size of the cell, and SSC—side scatter parameter referring to the observed cells’ granulation) were estimated by using the FACSVerse flow cytometer (Becton–Dickinson, Franklin Lakes, NJ, USA) for predefined amount of time (time-restricted acquisition of data) and standardized with results obtained for reference wells.

The monocytes’ FSC and SSC parameters changes after the exposure to bacterial biofilm as compared with the monocytes exposed to reference well—empty, sterile well were considered factors reflecting the activation of the monocytes upon the contact with bacterial biofilm (FSC/K, SSC/K). The adherence of THP-1 cells to the bacterial biofilm was assessed by measuring the number of cells in the tube for predefined amount of time, collected from the biofilm coated well after 1 h incubation on orbital shaker in comparison to the number of cells in the tube collected from the reference well—without biofilm (ADH/K).

### Data analysis

The differences were tested by analysis of variance (ANOVA) by StatSoft software (Statistica 10, USA).

## Results

### Specific characteristics of patients influence monocytes’ response to biofilm

**Fig. 1:**
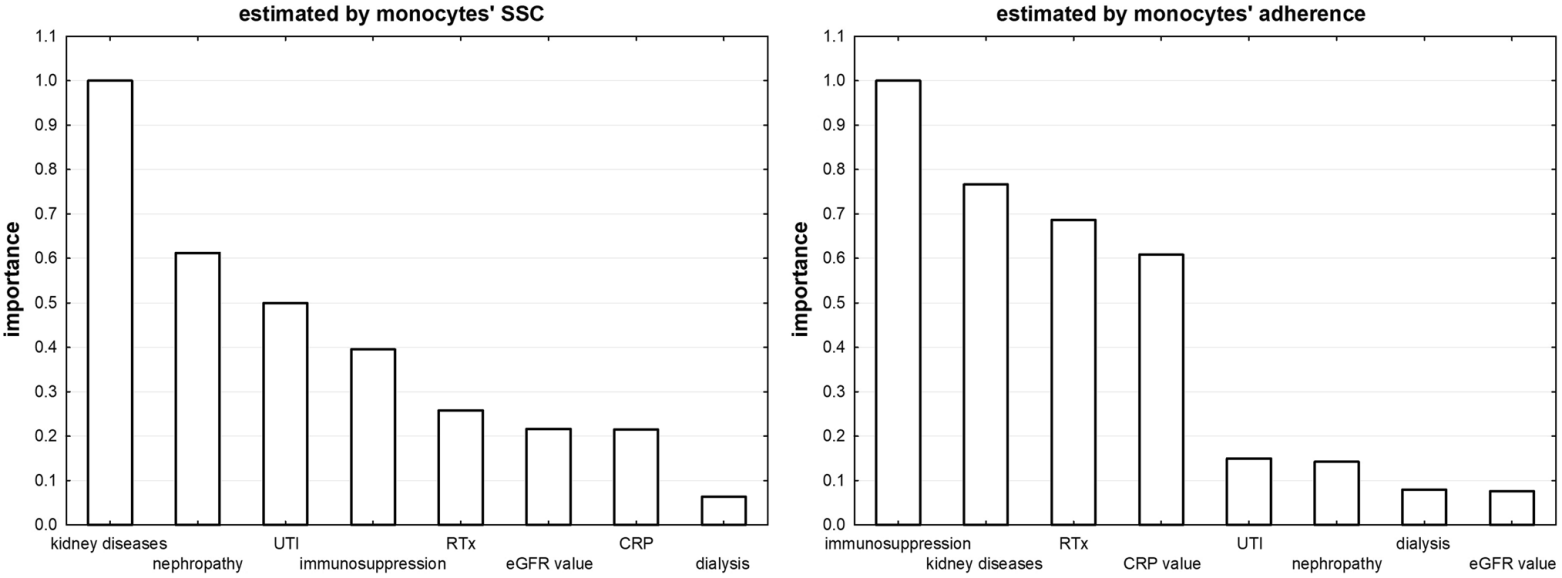
Various features of patients impact the monocytes response to biofilm exposure in different manner. *RTx* recipient of kidney’s transplant, *SSC* side scatter (granularity of the cell)

Interaction of monocytes with biofilm urine isolates of *E. coli* results in changes of monocytes’ morphology (described as FSC and SSC) and adherence to the formed biofilm depending the underlying disease of a patient. FSC value, which reflects the size of the cell, varied from 0.91 to 1.19 with median 1.02 and standard deviation 0.045. SSC (the cell’s granularity) median was 1.05 and varied from 0.88 to 1.66 with standard deviation of 0.1. Diversity of adherence was the most evident and varied from 0.02 to 8.98 with median 1.027 and standard deviation of 1.19. All used estimators (FSC, SSC and monocytes’ adherence to biofilm) are clearly modified by the type of kidney disease (importance factor 1 for FSC and SSC of monocytes, 0.75 for adherence) and profile of immunosuppression used in RTx patients (importance index 1 for adherence and FSC) as showed on Fig. 1. Hypertensive and diabetic nephropathy have slightly lower impact on modification of monocytes response by biofilm (importance index 0.6–0.8) and very low on adherence of monocytes. Additionally, an inflammation, measured as an elevation of CRP (C-reactive protein) level, significantly impacts both the adherence and FSC of monocytes.

### Adherence of monocytes to bacterial biofilm is influenced by many factors

**Fig. 2:**
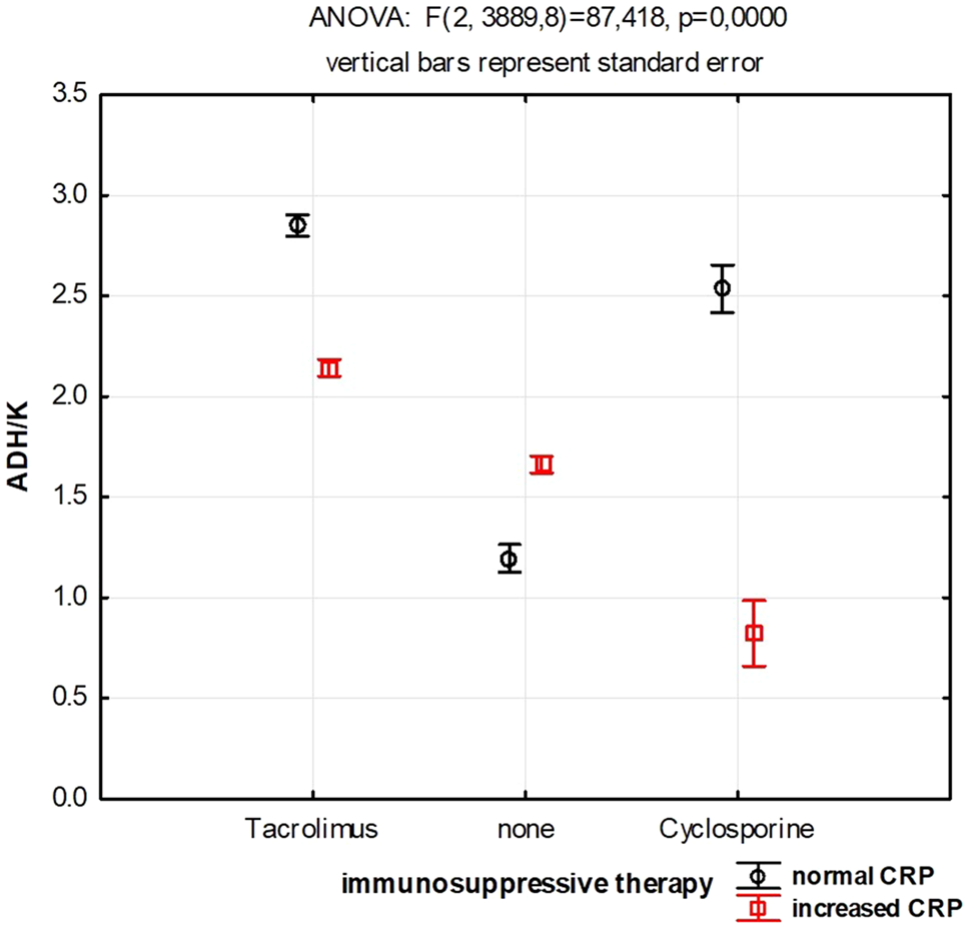
Immunosuppressive therapy influences monocytes’ response to biofilm. *ADH/K* monocytes’ adherence to biofilm in plate’s well normalized to the monocytes’ adherence to the well’s bottom without biofilm

Detailed ANOVA analysis shows higher adherence of monocytes to biofilm formed by isolates from patients undergoing immunosuppressive therapy without inflammation symptoms present (defined as CRP < 5ug/ml) at the moment of material obtaining. Additionally, along with an increase of CRP, the adherence of monocytes decreases when immunosuppression is present. The observed change is especially evident in case of patients treated with cyclosporine (Fig. 2.).

**Fig. 3:**
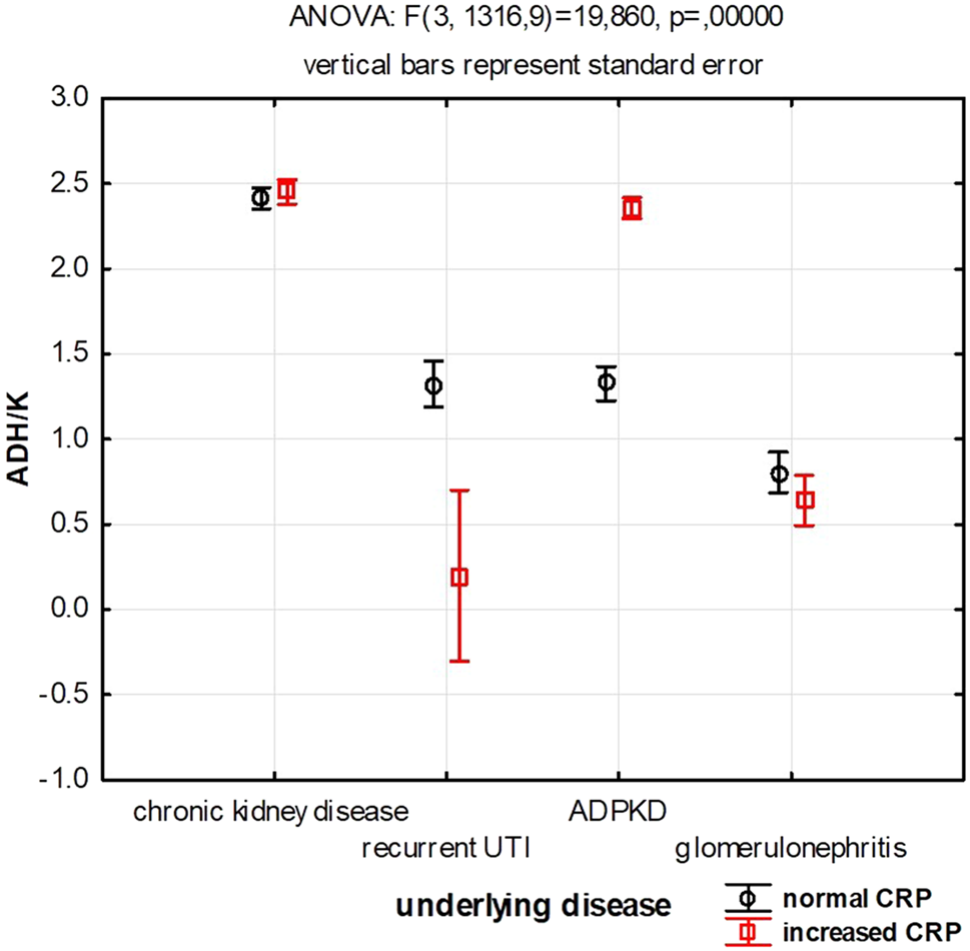
Monocytes’ response, measured as adherence to biofilm differs depending on the type of underlying disease. *ADPKD* autosomal dominant polycystic kidney disease; *ADH/K* monocytes’ adherence to biofilm in plate’s well, normalized to the monocytes’ adherence to the well’s bottom without biofilm

The adherence of monocytes is also modified by CRP and the type of kidney disease (underlying disease) (Fig. 3.). Isolates from patients with chronic kidney disease form biofilm against which monocytes show the strongest response, defined as their highest adherence to biofilm surface. The observation is the same regardless the presence or absence of inflammation. In case of patients with ADPKD (autosomal dominant polycystic kidney disease)—the presence of inflammatory reaction (measured by an increase of CRP) significantly increases the adherence of monocytes to the biofilm. On the other hand, in case of recurrent UTI (RUTI), completely opposite situation is observed: RUTI is characterized by lower adherence of monocytes to the bacterial biofilm when inflammation is present rather than when there is lack of inflammatory reaction.

### The changes of the size and granularity of the monocytes also depend on patients’ features

**Fig. 4:**
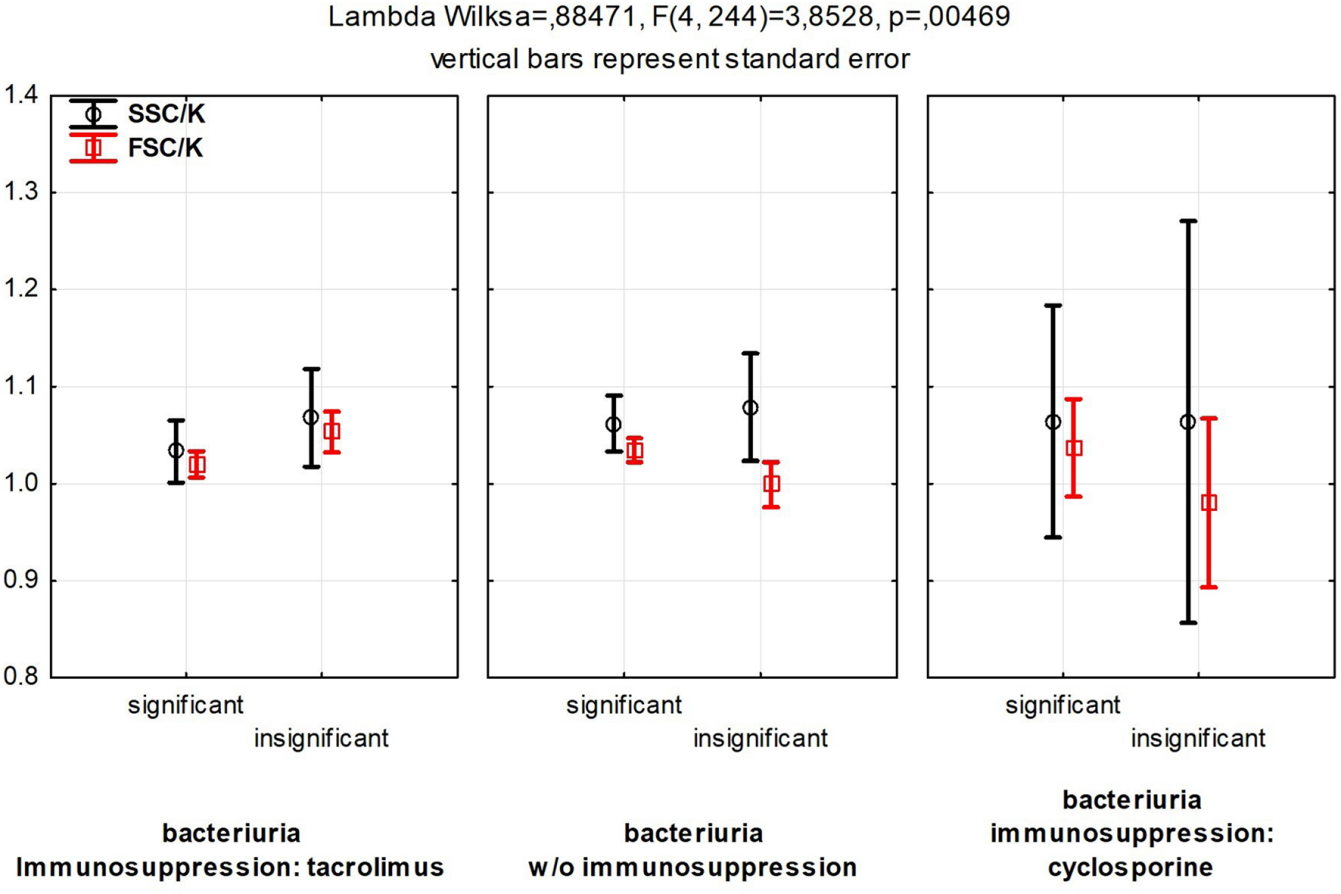
Monocytes’ response, measured as morphology changes, in response to biofilm depending on the type of immunosuppression and type of bacteriuria present. *SSC/K* granularity of monocytes exposed to biofilm normalized to the monocytes not exposed to biofilm. *FSC/K* the size of monocytes exposed to biofilm normalized to the monocytes not exposed to biofilm

Even though the size and granularity of monocytes do not change so drastically as their adherence to biofilm given the presence or absence of inflammation or the nature of underlying disease, biofilm of isolates from RTx patients causes more diverse changes in size (measured as FSC parameter) and granularity (measured as SSC parameter) of monocytes exposed to bacterial biofilm when the cyclosporine is used as an immunosuppressive drug (Fig. 4).

## Discussion

The majority of studied results advocate the importance of bacterial biofilm formation in UTIs, notably in chronic cystitis and infections associated with catheters. Surprisingly, some authors found that virulence seems to be inversely related to biofilm formation (Hancock et al. 2007). In the current study we evaluate the response of monocytes to formed biofilm. The host’s efficient control of invading pathogens to some degree strongly depend on pathogens properties, such as proliferation rate (Moreno et al. 2008) and resistance to phagocytosis (Groesdonk et al. 2006). However, it was shown recently that even bacteria that exhibit relatively low virulence, such as *Escherichia coli*, can induce cell death upon phagocytosis (Rowe and Juthani-Mehta 2014). Since underlying host factors, such as comorbidities, might affect urinary inflammation etiology, it is always worth to remember that the outcome of the inflammatory reaction depend not only on the bacteria properties but also on the host’s condition.

In one of our previous studies we have proved the difference in biofilm composition (e.g. protein level) and metabolic activity of biofilm formed by commensal and virulent strains (Meissner et al. 2013). Subsequently, higher adherence of monocytes was specific for the response to biofilm formed by bloodstream (invasive) isolates than to biofilm formed by urine (non-invasive) isolates (Jarzembowski et al. 2018). Different monocytes reaction to biofilm formed by RTx patient’s isolates was also observed when compared with isolates of the same origin from patients without immunosuppression (Jarzembowski et al. 2015).

The exposure of monocytes to bacterial biofilm results in their activation. It may be reflected by quick changes in their cytoskeleton (Mancilla-Herrera et al. 2015)—such reactions include changes in monocytes size (assessed in this paper by flow cytometer’s FSC parameter) and are not limited to bacterial infections only—such changes are also observed in viral infections—and it was reported that as the result, monocytes are actively secreting both pro- and anti-inflammatory cytokines, such as IL-6, IL-10 and TNF-α (Zhang et al. 2020) and engage in direct killing of the pathogens through oxidative and non-oxidative pathways, utilizing numerous granules they produce, such as myeloperoxidase, hydrolases and elastases, lysozyme and proteases (Koenig et al. 2017). All of that—cytokines production as well as enzymes engagement in oxidative burst—will in turn reflect the changes in monocytes’ granularity after activation—as observed after the exposure of monocytes to the biofilm. The increased granularity in itself is not surprising, because it reflects basic monocytes’ response to foreign element. The fact that there are measurable differences in the strength of the reaction when different immunosuppressive drugs are used and the significant or insignificant bacteriuria is present, reflects that there is a difference in biofilm composition and in bacteria selection in a patient body and those features influence immune system cells response (monocytes’ cell line in this case). To get a full picture of possible interactions and differences between the biofilm and monocytes (and further the innate immune system) the tests with monocytes isolated from the patients are planned.

Activation of monocytes by bacterial agents might be reflected also by other features, such as surface receptors repertoire (e.g. (Benoit et al. 2008; Italiani and Boraschi 2014; Strauss-Ayali et al. 2007; Imhof and Aurrand-Lions 2004)), and will be linked to both activation and migration with adhesion of the monocytes.

Gołębiewska et al. found that the prevalence of strains carrying various virulence genes seems to be affected by the choice of immunosuppressive regimen. This suggests that the type or strength of immunosuppression used, might influence the selection of strains with a particular virulence profile (Gołębiewska et al. 2019). Also our results illustrate high impact of the immunosuppressive therapy on the properties of biofilm and as the result, the monocytes’ response. In case of tacrolimus—regardless the presence or absence of inflammation, the adherence of monocytes is higher than when the immunosuppression is not present. In case of cyclosporine, the adhesion of monocytes increases only when there are no changes in CRP level and when the inflammation is present the observed adhesion is much lower. In case of cyclosporine maybe that is one of the reasons, why in the presence of immunosuppressive therapy the recurrence of UTI is higher than in its absence—by affecting the adherence and therefore the ability to kill bacteria directly by the monocytes using oxidative pathways. Biofilm eradication by the immune cells is harder than in case of planktonic cells, the monocytes adherence to the bacteria is crucial for the effective phagocytosis in both of them though. That of course is one of possible explanation. The main mechanism of action of cyclosporine is a direct effect on e.g. monocytes—through inhibition of TLRs (tool-like receptors), or direct inhibition of phagocytosis (Emal et al. 2019). Considering the fact that tacrolimus chemically belongs to the macrolides—antibiotics which have in their spectrum mainly Gram(+) cocci, no evident influence on Gram(-) rods could be expected. From the other hand, the immunosuppressive strength of cyclosporine is reported to be weaker than that of tacrolimus (Azzi et al. 2013).

Kidney transplantation is usually followed by immunosuppressive therapy to prevent rejection of graft. The calcineurin inhibitors represent the most commonly used agents. Several randomized trials have compared tacrolimus with cyclosporine to try and find the optimal agent for renal transplantation; however, studies have shown contradictory results. Cyclosporine seems to be significantly superior to tacrolimus in regard to diabetes but no significant differences between those two calcineurin inhibitors were found with regard to infection (Azarfar et al. 2018).

The etiology of the kidney disease was found to impact the properties of the biofilm formed by *E. coli* and *K. pneumoniae*. If the CRP value was increased, the adherence of monocytes to the formed biofilm was especially high in case of ADPKD and chronic kidney disease. In case of RUTI the presence of inflammation decreased adherence of monocytes, although the answer of monocytes was rather diverse there. ADPKD is the most common hereditary kidney disease. One of the most common complications of ADPKD are urinary tract infections (UTIs), with prevalence up to 60%. Unique properties of biofilm formed by isolates from ADPKD patients were also proved in our previous study of *ASA* gene expression (Daca et al. 2014). Enterococcal strains from patients with ADPKD differ from other end-stage renal diseases, taking into consideration biofilm formation. The isolates from urine of ADPKD patients have the tendency for relatively low level biofilm formation when compared with the ability of bacterial strains isolated from urine of patients with other renal diseases.

## Conclusions

As the adhesion was the factor differing the most in groups of patients, taking into consideration: (1) the presence or absence of immunosuppressive drugs and the presence or absence of inflammatory reaction, as well as (2) the underlying disease and the presence of inflammatory reaction we conclude that the differences in the structure of biofilm in those groups affect the behavior of monocytes to such degree that the measurement of monocytes’ adhesion to bacterial biofilm isolated from the patient may be considered as a new tool for individualized diagnosis of symptomatic and asymptomatic bacteriuria in the future if the situation requires such differentiation. Of course, careful in-depth analysis of underlying molecular and cellular causes of these differences will be required.

## Data Availability

Not applicable.
